# Are Heat Shock Proteins an Important Link between Type 2 Diabetes and Alzheimer Disease?

**DOI:** 10.3390/ijms21218204

**Published:** 2020-11-02

**Authors:** Joanne Elizabeth Rowles, Kevin Noel Keane, Thiago Gomes Heck, Vinicius Cruzat, Giuseppe Verdile, Philip Newsholme

**Affiliations:** 1 School of Pharmacy and Biomedical Sciences, Curtin Health Innovation Research Institute, Biosciences, Curtin University, Perth 6102, Australia; joanne.pradella@postgrad.curtin.edu.au (J.E.R.); kevin.keane@curtin.edu.au (K.N.K.); giuseppe.verdile@curtin.edu.au (G.V.); Philip.newsholme@curtin.edu.au (P.N.); 2 Physiology Research Group, Graduate Program in Integral Attention to Health (PPGAIS-UNIJUÍ/UNICRUZ) and Graduate Program in Mathematical and Computational Modeling, Regional University of Northwestern Rio Grande do Sul State (UNIJUÍ), Ijuí, RS 98700-000, Brazil; thiago.heck@unijui.edu.br; 3 Faculty of Health, Torrens University Australia, Melbourne 3000, Australia; 4 School of Medical and Health Sciences, Edith Cowan University, Joondalup 6027, Australia

**Keywords:** heat shock protein 70, IAPP, Aβ, iHSP, eHSP, dementia, tau

## Abstract

Type 2 diabetes (T2D) and Alzheimer’s disease (AD) are growing in prevalence worldwide. The development of T2D increases the risk of AD disease, while AD patients can show glucose imbalance due to an increased insulin resistance. T2D and AD share similar pathological features and underlying mechanisms, including the deposition of amyloidogenic peptides in pancreatic islets (i.e., islet amyloid polypeptide; IAPP) and brain (β-Amyloid; Aβ). Both IAPP and Aβ can undergo misfolding and aggregation and accumulate in the extracellular space of their respective tissues of origin. As a main response to protein misfolding, there is evidence of the role of heat shock proteins (HSPs) in moderating T2D and AD. HSPs play a pivotal role in cell homeostasis by providing cytoprotection during acute and chronic metabolic stresses. In T2D and AD, intracellular HSP (iHSP) levels are reduced, potentially due to the ability of the cell to export HSPs to the extracellular space (eHSP). The increase in eHSPs can contribute to oxidative damage and is associated with various pro-inflammatory pathways in T2D and AD. Here, we review the role of HSP in moderating T2D and AD, as well as propose that these chaperone proteins are an important link in the relationship between T2D and AD.

## 1. Introduction

Alzheimer’s disease (AD) and type 2 diabetes (T2D) are two of the most widespread age-related chronic diseases, and the prevalence of both is steadily increasing [1,2]. AD and T2D share similar risk factors, which can include a sedentary lifestyle, poor diet, obesity, and hereditary predisposition [3]. Studies have shown that patients with T2D are up to 65% more likely to develop AD than non-diabetic patients, while AD individuals are more likely to be insulin resistant [4,5]. T2D and AD also share dysfunctions in the insulin receptor, chronic inflammation, and secretion of amyloidogenic peptides [6,7].

Amyloidogenic peptides are peptides that spontaneously misfold, aggregate, and deposit in extracellular spaces, forming toxic soluble intermediates and insoluble fibrillar amyloid plaque. Amyloidogenic peptides are associated with the development of T2D and AD through the formation of islet amyloid polypeptide (IAPP) and β-amyloid (Aβ), respectively [8,9,10]. These two amyloidogenic peptides have similar methods of exerting toxicity involving membrane pore formation, mitochondrial dysfunction, oxidative stress, endoplasmic reticulum (ER) stress, and apoptosis [11,12]. While Aβ and IAPP deposit in their respective tissues of origin, they also co-localize in the plaque of both brain tissue and pancreatic islets, where they undergo misfolding and aggregation [13,14]. Once co-localized, Aβ and IAPP may also undergo a process called cross-seeding, where the aggregation and seeding of amyloidogenic peptides attract and aggregate with more similar and/or different types of amyloidogenic peptides [15,16]. In this case, co-localized Aβ and IAPP may promote the formation of combined Aβ-IAPP oligomeric hetero-complexes [17,18,19]. Such dysfunctions in the protein homeostasis of vital tissues often create a stressful environment in which cells may fail to thrive.

At the most basic cellular level, living organisms respond to stressful or unfavorable conditions by changing the expression of stress-related genes, predominantly via the transcription and upregulation of heat shock proteins (HSPs) [20]. This cytoprotective molecular organization is referred to as the heat shock response (HSR). HSPs are a class of proteins that are rapidly upregulated by cells in response to a variety of endogenous or exogenous stressors [20]. Despite being originally defined by their role in the thermal stress response [21], HSPs are now understood to be expressed both constitutively and in the presence of cellular stresses such as oxidative stress [22] and inflammation [22,23].

One of the main roles associated with the HSPs is to support protein maintenance, including assisting the proper folding of newly synthesized proteins, refolding and clearance of misfolded and/or aggregated proteins, and participating in the membrane translocation of secretory proteins [24]. As the folding, maintenance, and degradation of proteins are key requirements for cell homeostasis, alterations in the HSR and the associated functions of HSPs have been linked to chronic diseases, such as AD and T2D. In this review, we describe the potential roles for HSPs in AD and T2D pathogenesis.

## 2. Dysregulation of Cellular Homeostasis in T2D and AD

T2D is a chronic disorder characterized by the dysregulation of cellular metabolism, deposition of amyloid plaque, and disruption of insulin signaling. In T2D, the dysfunction of the insulin signaling mechanism results in the insensitivity to insulin in peripheral tissues, leading to hyperglycemia, hyperinsulinemia, and high levels of circulating lipids [25]. Complications of diabetes vary from acute, such as ketoacidosis and dehydration, to chronic complications, such as angiopathy, heart disease, kidney disease, neuropathy, and retinopathy [26]. Within pancreatic islets, T2D is associated with a reduced β-cell mass, inefficient glucose-stimulated insulin secretion (GSIS), and the deposition of aggregated IAPP as amyloid plaques [27,28].

IAPP has hormone functions under physiological conditions that assist in the regulation of post-prandial glucose levels [29]. IAPP is synthesized in the ER of β-cells from precursor protein ProIAPP and co-secreted with insulin at a ratio of 20 parts of insulin to 1 part IAPP [30]. As an amyloidogenic peptide, IAPP is prone to misfolding and aggregating into soluble oligomeric intermediates before progressing into insoluble fibrillar structures that deposit in the extracellular space of pancreatic islets as a plaque [28]. The soluble oligomeric intermediate species are the more toxic species, and unlike the insoluble fibrillar species, they have been identified both extracellularly and intracellularly [31]. Intracellular IAPP oligomers may contain ProIAPP and can be present throughout the secretory pathway, including the ER, secretory vesicles, and cytosol, indicating that misfolding and aggregation is possible prior to secretion with insulin [32,33]. In addition, IAPP may be secreted as monomers, aggregate externally, and then re-enter cells via micropinocytosis or independently cross membranes in a similar fashion to cell-penetrating peptides [34,35]. Whether external or internal, IAPP is capable of forming inappropriate ion channels in cell membranes, such as mitochondrial and plasma membranes, leading to intracellular ionic dysregulation and mitochondrial dysfunction [12,33]. Mitochondrial dysfunction is not only detrimental to β-cell viability but drastically impairs insulin secretion [36,37]. The reduction in β-cell mass, mitochondrial dysfunction, and decreased GSIS potentiate the toxic effects of amyloidogenic peptides during the course of T2D.

**Figure 1:**
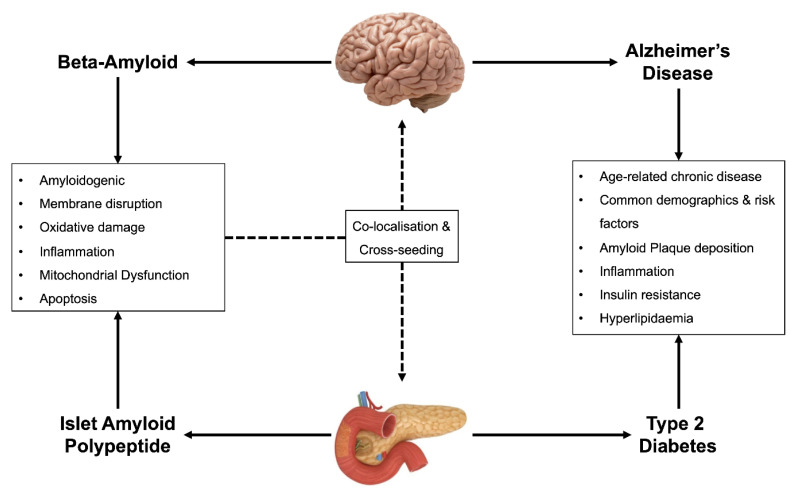
Association between Type 2 diabetes and Alzheimer’s disease. Type 2 diabetes (T2D) and Alzheimer’s disease (AD) are both age-related chronic diseases, affecting the similar populations of people. Both diseases also feature altered metabolism, dysfunctions in insulin signaling, and plaque deposition composed of amyloidogenic peptides such as islet amyloid polypeptide (IAPP) and β-amyloid (Aβ). Similar to the diseases themselves, IAPP and Aβ share many commonalities, which are predominantly shared mechanisms of toxicity. Current research suggests that IAPP and Aβ can co-localize in the brain and pancreatic islets and cross-seed to form IAPP–Aβ heterocomplexes with potentiated toxicity.

Similarly, to T2D, AD is a chronic disorder associated with the dysregulation of cellular metabolism, dysfunctional insulin signaling, and the deposition of insoluble plaque in vital tissues. The association between T2D and AD is shown in Figure 1. AD is a progressive neurodegenerative disease leading to memory loss and an eventual loss of psychomotor skills and control of bodily functions [38]. The brains of AD patients often show amyloid plaques composed of Aβ, as well as neurofibrillary tangles, consisting of hyperphosphorylated tau (pTau) protein [39]. In addition, neuroinflammation has also long been considered a key feature of the AD process [40], and recent evidence supports the theory that that the presence of Aβ plaques initiates the inflammatory process and promotes tau accumulation [41,42,43]. A number of mechanisms underlying neurotoxicity in AD have been proposed, including metabolic dysregulation and mitochondrial dysfunction. Similarly to IAPP, Aβ may interact directly with membranes to form unregulated ion channels [44]. The dysregulation of ions within the cell, or within specific organelle membranes such as the mitochondria, can result in alterations in mitochondrial membrane potential and unbalance in the redox state of the cell, leading to oxidative stress [45,46].

Another important physiological connection between T2D and AD involves the impairment of insulin signaling in the central nervous system (CNS). Insulin signaling is strongly associated with memory and learning in the CNS, but it may also affect Aβ production, Aβ clearance, and tau phosphorylation [47]. The presence of insulin resistance in peripheral tissues, as noted in T2D, potentially also reflects the presence of insulin signaling dysfunction throughout the CNS, offering a possible explanation for the close association of T2D and AD. It has been noted that insulin signaling is downregulated in AD-affected brains and that Aβ itself may bind to the insulin receptors, impacting synaptic strength and raising the possibility that Aβ could competitively inhibit the binding of insulin [48]. Additionally, both Aβ and IAPP have demonstrated an ability to inhibit insulin sensitivity and glucose uptake in peripheral tissues, primarily via the phosphorylation of Serine 473 of Akt [49,50,51].

Amyloidogenic peptides have an innate ability to recruit monomeric peptides and incorporate them into the growing aggregated complexes [52]. This process, referred to as “seeding”, starts with a progressive nucleation-dependent phase where an aggregated seed or “nucleus” forms. Once formed, the rapid elongation phase begins as the recruitment of monomeric proteins is added to the aggregated mass due to thermodynamically favorable interactions, forming insoluble complexes [53]. As the intermediate and end products of all amyloid aggregations have common β-sheet structures, different amyloids such as IAPP and Aβ have been shown to actively interact and form hetero-oligomer and hetero-fibril complexes [17,54].

IAPP can function as a seed for the aggregation of Aβ and potentiate Aβ toxicity [45,55]. These complexes increase in cytotoxicity in the presence of increasing concentrations of Aβ and/or IAPP [54]. This could particularly impact tissues where Aβ and IAPP are predominantly produced and may then co-localize, such as the brain and pancreatic islets. Recent studies indicate that IAPP may travel systemically from the pancreas and cross the blood–brain barrier (BBB) to deposit with Aβ plaque in the brain [14,56]. Co-oligomerized IAPP–Aβ complexes can increase neuronal cell death up to 3-fold compared to similar concentrations of IAPP or Aβ alone [17]. Interestingly, peripherally produced amylin can bind to amylin receptors in the endothelial cells of the BBB, increasing the translocation of Low-density Lipoprotein Receptor-Related Protein 1 (LRP1) to the cell membrane and thereby actively promoting the transport of brain Aβ into the blood [57]. While transporting soluble Aβ from the brain to the blood has the benefit of reducing toxic Aβ species from the brain, it is in lieu of degradation pathways and does not decrease brain plaque burden [58]. Then, peripheral insulin-sensitive tissues, such as skeletal muscle and liver, are exposed to toxic-soluble Aβ species that may reduce insulin sensitivity [52]. Aβ can also travel systemically to the pancreatic islets, although evidence also suggests that the pancreatic islets may be capable of the endogenous production of Aβ [13,56,59]. Similar to the brain, co-oligomerized IAPP–Aβ complexes exert enhanced toxicity to cells in the pancreatic islets than either IAPP or Aβ alone [52].

Considering both the critical role for HSPs in protein folding and that they are expressed in response to misfolded or aggregated proteins, it stands to reason that these molecular chaperones have important roles as a response to aggregated Aβ/Tau in AD or IAPP in T2D. Indeed, there is mounting evidence that the HSF and a number or members within the HSP family are activated in the presence of these amyloidogenic proteins and other stresses associated with AD and T2D.

## 3. Activation and Characterization of Heat Shock Proteins

The ability of all living organisms to respond with rapid and appropriate modifications against physiological challenges is an essential feature for survival. The expression of HSPs is the most highly conserved genetic system against cellular stress, and it is present in almost all known organisms [60]. The predominant role of the HSP family of proteins is to protect cells and facilitate the recovery of disturbed metabolic pathways [61]. There are a number of members of the HSP family that are named according to the gene that encodes them (e.g., HSPA1A, HSPA8 genes) or by the apparent molecular weight in kDa (e.g., 70 kDa HSPs as HSP70).

In humans, the HSR is primarily regulated by heat shock factors (HSFs), which are transcriptional activators of the HSP genes. While inactive, HSF monomers bind to HSPs, forming an inactive complex. As protein misfolding commences, HSPs begin dissociating themselves from HSFs and migrate to the misfolded proteins. This allows the HSFs to undergo trimerization and translocate to the cell nucleus. Here, they bind to heat shock elements within the promoter regions of their target genes and activate the transcription of new HSPs [62]. Although there are four identified HSFs, only HSF1, HSF2, and HSF4 have been identified in humans. HSF2 and HSF4 are not known to be extensively involved in the HSR and have been suggested to be predominately involved in embryonic development and tissue-specific transcriptional regulation, rather than the HSR. Traditionally, HSF1 is known as the “master regulator” and is both the best characterized and most intimately involved in the HSR [63,64,65,66]. Interestingly, recent evidence suggests that HSF2 may regulate HSF1 activity via the formation of heterotrimers, altering HSF1′s responsiveness to stress [67]. Regardless, once activated and transcribed by HSFs, the HSPs begin their role as molecular chaperones.

HSP70 is the most characterized member of the HSP family, the members of which also includes HSP90, HSP60, HSP40, and the lower molecular weight species known collectively as small HSPs (sHSP) [68,69]. Apart from their size, key differences in functionality also separate the HSP families. For example, the higher molecular weight species (HSP90-40) are ATP-dependent in function, requiring ATP hydrolysis to alter their conformation to become active, while the sHSPs are ATP-independent and only activated by cellular stress [69]. Differences in subcellular locations are also common, as some HSPs only affect specific organelles (such as the mitochondria or ER), while others are cytosolic [61,68].

Although the HSR is a vital mechanism for homeostasis, HSPs are not only produced during cellular stress. Some HSPs are known to be constitutively expressed, with essential roles in cellular functioning that include controlling the trafficking of proteins in the cytosol and across cell membranes, the folding of recently synthesized proteins, and assisting in the assembly of large protein complexes [69,70]. HSPs can also be located in the extracellular spaces (termed eHSPs). In contrast to the classical anti-inflammatory and protective roles of intracellular HSPs (iHSP), eHSPs are thought to have a pro-inflammatory role, particularly in age-related chronic diseases [71,72]. Discussed further below in this review is the role of eHSPs and the importance of maintaining an appropriate balance between the levels of iHSPs and eHSPs for cellular homeostasis under conditions of stress.

## 4. Heat Shock Response in Type 2 Diabetes and Alzheimer’s Disease

**Figure 2:**
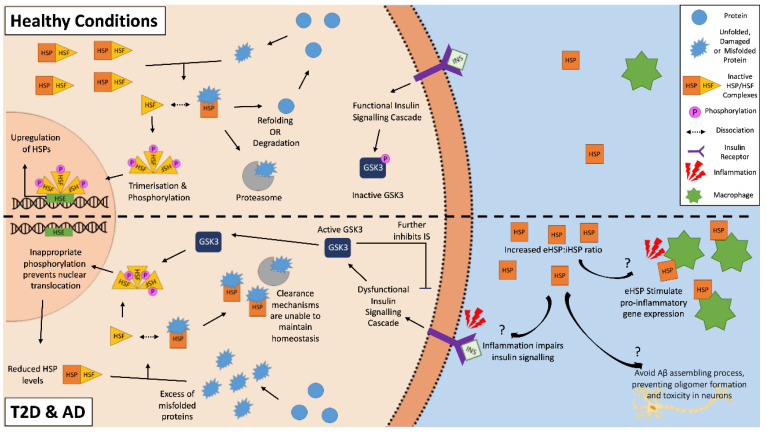
The heat shock response in Alzheimer’s disease and type 2 diabetes. The inflammatory environment in AD and T2D negatively disrupts insulin signaling, activating glycogen synthase kinase-3 (GSK-3), which in turn inappropriately phosphorylates heat shock factor 1 (HSF1). This phosphorylation inhibits the translocation of the HSF1 trimers to the nucleus, and as a result, inhibits the upregulation of HSPs. This reduced intracellular HSP pool is unable to effectively clear the aggregated amyloidogenic peptides within the cells. In the external environment, increased levels of extracellular HSPs found in T2D and AD patients can act as “chaperokines” and stimulate the immune system to produce pro-inflammatory factors. This becomes a vicious cycle of increased inflammation, decreased insulin signaling, and a decreased ability for HSP to clear aggregated peptides. On the other hand, some eHSPs (e.g., eHSP90 and eHSP70) in AD may have a role in the clearance of amyloid plaque, as they can modulate Aβ toxicity.

HSPs and the HSR have a predominantly cytoprotective role with the potential to attenuate T2D or AD pathology. HSPs can clear aggregated amyloid proteins and prevent further amyloid aggregation by inhibiting both the nucleation and elongation processes of cross-seeding [73,74]. However, both AD and T2D may feature an altered expression of HSPs, as the HSR is often dysregulated in aged and obese individuals, and phenotypes often seen in both of these two chronic metabolic-associated age-related diseases [75,76]. A downregulation of HSP and the HSR correlates with dysfunctional insulin signaling, which is another feature of both diseases, suggesting a strong correlation between HSPs and insulin signaling [77,78,79]. A potential reason for this involves glycogen synthase kinase-3 (GSK-3), which is a negative regulator of the insulin signaling cascade. An inflammatory environment resulting from chronic disease and obesity, such as in AD and T2D, negatively impact insulin signaling, which results in the activation of GSK-3 [79,80]. As well as further impairing insulin signaling, activated GSK-3 can phosphorylate HSF1 [81,82]. The phosphorylation of HSF1 inhibits its translocation to the nucleus, thereby lowering the gene expression of HSPs. The inhibition of GSK-3 causes an upregulation of HSP and restoration of insulin signaling [79,81,83,84]. Then, a vicious cycle can be established, where the inflammatory environment impairs insulin sensitivity and insulin signaling, which in turn impairs the cells’ ability to manage the stresses of the local environment via the downregulation of HSPs, making insulin-sensitive tissues more susceptible to damage and resulting in further increases in inflammation and hyperglycemia (Figure 2) [79].

In neuronal tissue and pancreatic islets where amyloid plaque can accumulate, impaired insulin signaling can result in a low HSP environment, favoring an increased aggregation of amyloidogenic peptides [82]. However, in a T2D monkey model, it was shown that despite decreases in HSP and HSF levels in peripheral tissues such as the liver, HSF1 expression was increased, and HSP levels were maintained in pancreatic tissue [85]. This could indicate a possible islet-specific mechanism for protection from the inflammatory environment compared to peripheral tissues at early stages of T2D.

It has also been suggested that decreased membrane integrity could contribute to the reduction of HSP and HSF levels. Membrane lipid defects affecting integrity, fluidity, and composition, are featured in both AD and T2D [86,87]. HSPs are known to mediate membrane integrity, supporting the cell during stressful conditions [78]. Therefore, the combination of a reduction in HSPs and lipid defects induced by insulin insensitivity could further potentiate lipid membrane disruptions [88]. Considering that that there are several HSP proteins and that they have different locations and functions, the effects on the pathology in T2D and AD cannot be generalized amongst all members of the HSP/HSF family. Indeed, evidence to date implicate HSP90, 70, 60, and 40 in moderating Aβ, Tau, or IAPP aggregation, or cellular stress induced by these aggregated proteins [89]. There are also emerging roles for sHSP and extracellular eHSP in T2D and AD. These topics are discussed further in the present review.

### 4.1. Heat Shock Protein 90

HSP90 is the most abundantly expressed HSP protein in eukaryotic cells [90]. Predominantly located in the cytosol, the functions of HSP90s include mediating the inflammatory response, as well as stabilizing and correcting misfolded proteins [90]. Experimental evidence suggests that HSP90s are involved in regulating the activity of several signaling proteins, such as steroid hormone receptors, and cellular differentiation processes [89].

Although the expression of HSP90 is predominantly constitutive, it can also be stress-induced in response to misfolded or aggregated proteins. HSP90 can inhibit Aβ toxicity by binding misfolded Aβ peptides [91]. Once bound, HSP90 then prevents further aggregation using an ATP-independent pathway or by changing the conformation of Aβ to a state less prone to aggregation via an ATP-dependent pathway [91]. In addition, tau is a substrate protein for HSP90 chaperones, where they can bind hyperphosphorylated tau and activate degradation processes [92,93]. As for IAPP, evidence shows that the ubiquitin–proteosomal system, which includes HSP90, is important for IAPP clearance and turnover. A decline in the function of this system due to inflammation and aging is detrimental to pancreatic islets, allowing IAPP aggregation to occur [94,95,96].

The role of HSP90 in T2D and AD is complex, as HSP90 can reportedly have both protective and detrimental roles in managing amyloidogenic peptides. The inhibition of HSP90 promoted an increased clearance of Aβ and tau in primary neuronal cells of rats [97,98], and it also facilitated better glucose regulation in T2D mice [99]. HSP90 inhibition also increased the HSR by increasing the dissociation of HSF1 from HSP90, where it could translocate to the nucleus and activate/potentiate the HSR [97]. HSP90 assists in the maintenance and function of GSK-3 [98], which is well known for its detrimental effects in T2D and AD [81,84]. In addition, HSP90 may even facilitate tau aggregation and hyperphosphorylation in rat brain extracts by promoting conformation changes in tau that promote its phosphorylation by GSK-3 [100].

As HSP90 actions can vary so widely, post-translational modifications and co-chaperones are among the most important factors in the regulation of HSP90 activity. This includes the acetylation and/or phosphorylation of HSP90, as well as the formation of larger protein complexes with other HSPs, particularly HSP70 and HSP40 [101]. The HSP90/70/40 complex can slow Aβ aggregation in a chaperone dose-dependent manner. In addition, in brain tissue from both transgenic AD mouse models and AD patients, expression or formation of the HSP90/70/40 complex has been found to inversely correlate with tau aggregation [102], supporting a protective role for this complex.

### 4.2. Heat Shock Protein 70

The HSP70 family is a varied group of chaperones with wide-ranging functions and subcellular locations. A defining feature of the group, aside from their similar molecular weight, is the shared structure of a substrate binding domain at the C-terminus that bind polypeptides, and at the N-terminus, a nucleotide binding domain (NBD) that interacts with ATPase to hydrolyze ATP [103]. Members of this family include the constitutively expressed Heat Shock Cognate 70 (HSC70) and the stress-induced HSP72, which are both found in the cytosol. Other members of the HSP70 family are found in organelle-specific locations, such as glucose-responsive proteins 78 (GRP78) and 75 (GRP75) are localized in the ER and mitochondria, respectively [104].

HSC70, as a constitutive HSP, is involved in general proteostatic functions, such as the support of protein assembly and protein trafficking throughout the cell. It also has roles in the innate immune response, as well as cell differentiation processes [69,105]. However, during cellular stress, HSP72 is one of the most strongly induced chaperones in the HSR and is considered one of the main stress-responsive chaperones in cells [106].

In T2D patients, HSP72 has been reported to inhibit the aggregation of IAPP [107,108]. Heat therapy to induce HSP72 was found to reduce insulin resistance and improve clinical parameters in T2D patients [78]. This was suspected to result from an HSP72-induced reduction of pro-inflammatory signaling molecule phosphorylation, which impaired normal insulin responses [78]. HSP72 can increase the fatty acid oxidation capacity in skeletal muscle, protecting against increases in insulin resistance and body weight [108]. Furthermore, HSP72 mRNA expression in skeletal muscle correlates with mitochondrial enzyme activity, rate of lipid turnover, and insulin-stimulated glucose uptake [77,109]. However, skeletal muscle HSP72 mRNA expression has been shown to be reduced in T2D patients compared to controls, suggesting a less efficient HSR [77]. In addition, HSP70 can be susceptible to glycation in a hyperglycemic environment, further reducing its chaperone activity [110].

In AD, the HSP70 proteins have cytoprotective roles via different mechanisms, including the inhibition of Aβ oligomerisation and remodeling to a less amyloidogenic form [90,92], upregulation of Aβ degradation enzymes [89,111,112], and restoring tau homeostasis by promoting the degradation of pTau aggregates, most likely by the ubiquitin–proteasome and/or autophagy systems [89,112]. However, HSC70 and HSP72 had opposite effects on tau stability; HSP72 increases the degradation of tau, while HSC70 reduces it. The ratio of inducible to constitutive HSPs appears to be a critical factor, as increased levels of HSP72 appears to negate the ability of HSC70 to stabilize Tau [113,114].

Other members of the HSP70 family, such as GRP78, also have documented roles in AD pathology. Intracellular Aβ oligomers can cause cellular damage before becoming extracellular. To prevent Aβ intracellular toxicity, GRP78 can bind to Aβ precursor proteins in the ER, preventing the β/γ-secretase cleavage necessary to process APP to Aβ, as shown in an HEK cell model co-transfected with APP and GRP78 [115].

### 4.3. Heat Shock Protein 60

HSP60 chaperones are traditionally associated with the mitochondria, and various studies have indicated that HSP60 may be more ubiquitously expressed than previously thought [116,117]. Within the mitochondria, HSP60 works closely with co-factor HSP10 (a member of the sHSP family) to maintain and correct the folding of mitochondrial proteins. If a deficiency in these HSPs were to occur, as observed in skeletal muscle of T2D patients [118] and the cortex of AD patients [119], cellular stress is enhanced [89].

Neuroprotective effects of HSP60 have been demonstrated in a human neuroblastoma cell line, where the overexpression of HSP60 inhibited an Aβ-induced reduction of Cytochrome C Oxidase (COX) IV activity in the mitochondria, subsequently reducing apoptosis [117,120]. However, HSP60 has also been implicated in pro-apoptotic functions in cells. HSP60 was shown to bind to pro-caspase 3 in vitro and accelerate its maturation during apoptosis [89,121,122]. Furthermore, HSP60 can mediate the mislocalisation of APP to the mitochondria, where Aβ peptides can aggregate, possibly leading to mitochondrial dysfunction [123]. Considering these somewhat opposite effects reported for HSP60 on AD pathology, further investigation is warranted, potentially examining the role of expression or interactions with other HSPs that moderate the overall activities of HSP60.

### 4.4. Heat Shock Protein 40

The HSP40 family of chaperones function differently compared to other HSPs, as they require co-chaperones from the HSP70 group to be active [89,124]. HSP40 uses a conserved N-Terminus J-Domain to bind to the ATPase N-Terminus of HSP70s, stimulating ATP’s conversion to adenosine diphosphate (ADP) via hydrolysis. Once this occurs, HSP70 becomes activated, dissociates from HSF1, and begins binding to non-native proteins [125]. HSP40 may also bind to substrates and escort them to the substrate-binding domain of HSP70, where they mediate the process of refolding the substrate proteins. This HSP70/40 complex greatly enhances the efficiency and capability of the refolding cycle, including increasing ATP hydrolysis rate up to 1000-fold over basal levels [124,126].

While HSP40 is not directly involved in the pathogenesis of T2D or AD, it can facilitate and regulate many of the HSPs that are—for example, the HSP70/40 complex, which has been shown to inhibit Aβ aggregation in neuronal cells [102]. Interestingly, the B3 member of the HSP40 co-chaperone family, along with HSP72, has been shown to mediate glucose uptake and insulin signaling via c-Jun N-terminal kinase (JNK) repression [127]. Thus, alterations in the expression or function of HSPs in tissues associated with amyloidogenic peptides could result in the promotion of chronic disease such as T2D and AD.

### 4.5. Small Heat Shock Proteins

sHSPs are a family of ATP-independent chaperones with sizes ranging from 10 to 40 kDa that share common features, including a conserved α-crystallin domain. This domain allows for dimerization, leading to oligomeric assembly of the sHSPs, where they can then successfully bind non-native protein substrates and form stable sHSP–substrate complexes [89]. The oligomeric complexes are known for binding to several non-native proteins at once, which is a feature that is absent in other molecular chaperone families [128,129]. ATP-dependent chaperones are required to release the non-native protein from the sHSP–substrate complex, where it can then be refolded or degraded. By creating a reservoir of proteins for refolding or degradation, the presence of sHSPs makes the process more efficient [128].

sHSP have vital roles in T2D and AD. The expression of sHSPs is upregulated in T2D-associated tissues, including skeletal muscle, retina, and cardiac muscle. However, their ability to function correctly is often negatively affected, as both the solubility and activation of specific sHSPs is reduced in hyperglycemic environments [130,131]. In AD, the HSP27 species of sHSP preferentially interacts with hyperphosphorylated tau in human brain samples. In vitro, HSP27 modulates tau pathology by decreasing the level of hyperphosphorylated tau, suppressing tau-induced apoptosis and increasing the amount of dephosphorylated tau [132]. Furthermore, sHSP species can directly bind to Aβ and IAPP to prevent fibril formation [133] and aggregation. Aβ binding to metal ions, such as Cu^2+^, forms metal–peroxidases, which contribute to oxidative stress and an increased aggregation of Aβ. sHSP chaperones have been shown to prevent the Cu^2+^-induced aggregation by dislodging the bound Cu^2+^ ion, preventing fibril formation [134]. However, preventing fibril formation may not result in reductions in amyloid-induced toxicity. When the sHSP, αB-crystallin, was co-incubated with Aβ peptides in vitro, it inhibited the aggregation of Aβ into fibrils and instead maintained the Aβ peptides in oligomeric αB-crystallin/Aβ complexes that were more toxic to neuronal cells [135].

## 5. Extracellular HSPs

Since the discovery of HSPs in 1962, several in vitro and in vivo studies have demonstrated that an increase in intracellular HSP (iHSP) is an important mechanism for cell protection and survival against cell stress challenges [136]. However, subsequent studies over the past 30 years have shown that eHSPs are involved with a more complex signaling network with various biological, homeostatic, and immunomodulatory properties.

HSPs are exported to the extracellular space through two main different mechanisms: active, due to a nonconventional secretory process, and passive, which is secondary to cell death and lysis [137]. Briefly, an active mechanism (also known as non-classical or unconventional secretory pathway) occurs through the lysosome–endosome pathway where HSPs, such as HSP70, are translocated into the lysosome lumen via an ATP-binding cassette (ABC) transport-like system and further transported outside the cell via the endocytic process or secretory-like granules. The most accepted mechanism for the release of HSPs into the extracellular space is via extracellular vesicles derived from the plasma membrane (called exosomes) mainly through endocytosis [138,139]. However, apart from exosome release [140], there are other potential mechanisms that have been proposed by which eHSP is released, including via lipid rafts interactions [90,141], α1-andrenergic receptor-mediated pathways [142], and upon necrotic or apoptotic cell death [143,144].

Similar to the iHSPs, eHSPs are involved in protein folding quality control. Interestingly, eHSPs, such as eHSP90, eHSP70, eHSP60, and eHSP27 might be highly expressed and released as “chaperokines”, where they play a major role in inflammation and immunity [142,145,146]. However, dependent on the circumstances, the same HSP proteins are not as highly expressed and can be observed in lower amounts in the extracellular space, such as eHSP60 [147] and eHSP27 [148]. eHSPs can stimulate inflammatory cytokine release from immune cells [141,143], recruit Natural Killer (NK) cells [141], and activate microglial phagocytosis [70]. eHSPs also facilitate innate immune responses by binding to Toll-like receptors (TLRs), most likely TLR2 and TLR4, and activating the inflammatory cascade mediated by NF-κB [139,140,142,149]. A combination of oxidative stress and high levels of eHSPs, such as eHSP70, can also worsen the prognosis of inflammatory processes that occur in chronic diseases [150], such as in T2D [139] and AD [144]. eHSP are more susceptible to oxidative stress in the extracellular space, such as the bloodstream [138]. For instance, the increased protein oxidation in serum observed in AD [144] was associated with the functional impairment of HSP70 [151]. Moreover, oxidized protein aggregates may remain in extracellular fluids in the form of soluble aggregates and stimulate neutrophils to produce high levels of ROS and thereby promoting oxidative stress [27] and the activation of immune-inflammatory responses [152].

In T2D, iHSP levels (e.g., iHSP70) are decreased, while eHSP levels are increased and positively correlated with the progression of the disease [139,153]. eHSPs in T2D patients, such as eHSP70 and eHSP60 [154], are correlated with insulin resistance and beta-cell dysfunction and death [155]. On the other hand, eHSPs do not necessarily have a pro-inflammatory role under all circumstances. For example, eHSP90 may have a role in the clearance of amyloid plaque, as they can modulate Aβ toxicity in AD [156] and upregulate its clearance via the stimulation of TLRs and microglial phagocytosis [157]. Additionally, eHSPs may even have protective roles against protein aggregation, since eHSP70 may affect the Aβ assembling process, preventing oligomer formation and toxicity in neuronal cells in vitro. Thus, there is an inverse relationship between the presence of HSPs (mainly HSP70), the stage of Aβ oligomers, neurotoxicity, and the incidence of AD, as the expression and circulating levels of HSPs decrease with aging [152].

Thus, evaluating eHSPs levels outside the cells (e.g., bloodstream or liquor) might be useful to understanding the nature of the HSR as a biomarker of defense integrity of the organism. If we consider that iHSPs act in recovering homeostasis by inducing eNOS-dependent NO production, activating antioxidant enzymes, and inactivating NF-ΚB, contrarily, eHSPs can induce the oxidative inflammatory process by binding to NF-ΚB and AP-1, thereby inducing the expression of enzymes involved in ROS production, adhesion molecules, and in the release of inflammatory interleukins [158]. In addition, the same metabolically stressful situations that trigger the HSR within the intracellular milieu are able to activate the release of exosomes containing eHSPs by non-canonical secretory mechanisms. eHSPs can bind to membrane receptors (e.g., Toll-like) bringing about pro-inflammatory cytokine-like signals toward all tissues for the presence of homeostasis-threatening condition. However, at the same time that T2D patients (and other cardiovascular diseases patients) present a chronic low-grade inflammatory profile with an increase in the eHSPs levels, the HSR is impaired in these patients and associated with oxidative stress (a characteristic common in AD and T2D). Consequently, the eHSPs, even at higher levels in the bloodstream, have poor immune signaling properties after oxidation. This scenario represents a suppressed anti-inflammatory HSR, which may be a permissive situation for AD and T2D development [159].

Considering the “good” and “bad” roles of HSPs in the intra and extracellular milieu, as a whole, the balanced ratio between iHSP and eHSP in order to prevent cytotoxic effects of eHSP has been proposed [160]. In addition, a better understanding of the role of the eHSP and eHSP/iHSP ratio may contribute to the development of novel opportunities for the prediction, identification, diagnosis, management, and treatment of chronic diseases, such as T2D and AD.

## 6. Conclusions: Heat Shock Proteins as a Therapeutic Target

In this review, we have identified the upregulation of HSPs as thermally activated therapeutic targets for the treatment of chronic age-related diseases such as T2D and AD (see above). HSPs are a collective family of proteins that are suffixed by their molecular mass (in kilodaltons; kDa), both constitutively expressed, and inducible isoforms across several intracellular tissue sites and in extracellular fluid following stress [20]. Compared to increased intracellular HSP content (a necessary component for protective cellular adaptation), the presence of extracellular changes in HSP concentration reflects a relatively lower transient stress response which may act acutely as a signaling response. The 70 kDa and 90 kDa families of HSPs, normally known as HSP70 and HSP90, are generally the most widely studied responders to thermal stressors. Chaperones ensure appropriate cell function in a wide variety of conditions and have distinct roles in several physiological adaptations, including the unfolded protein response, e.g., recognizing misfolded or mislocalized proteins that may be subsequently degraded by the proteasome, and are a key component of chaperone-mediated autophagy [161,162]. We have described each of these roles above. The reader is directed elsewhere to contextualize these actions.

As therapeutic targets, HSPs may be considered to have a direct and indirect role in chronic diseases associated with the aggregation of misfolded proteins, including AD and T2D. In AD, HSP70 may suppress the proteolysis of Aβ precursor proteins [163] and in addition to HSP70, HSP90, and small HSPs reduce the formation of Aβ fibrils and Aβ toxicity [164]. Tauopathy occurrence in AD may also be positively impacted by HSP changes in HSP70 and HSP90 [165]. In a similar manner, HSP70 also reduces the formation of amyloid fibrils by preventing the primary nucleation and aggregation of misfolded IAPP [107]. In light of this, HSR inducible medications are suspected of having therapeutic potential in the treatment of these diseases. For example, geranylgeranylacetone (GGA), BGP-15, and Matrine have all shown some ability to promote glucose tolerance and insulin sensitivity in peripheral tissues, improving diabetic outcomes [166,167,168]. Matrine has also shown an ability to reduce the pro-inflammatory actions of microglia in the brain, potentially affecting the progression of AD [147,169]. Other methods of HSR induction, such as heat therapy, exercise, and mild electrical stimulation, have also shown therapeutic potential in the treatments of T2D and AD [167,170,171,172]. Despite the body of evidence available demonstrating the role of HSPs as therapeutic targets in T2D and AD, this is still an area with large potential to explore.

Much of the literature describing these responses involve complex and isolated tissue/cell models to understand how HSP manipulation impacts upon amyloid-associated disease factors; thus, direct application for humans remains undefined. However, with mechanistic support for the role of HSP augmentation to improve disease outcomes, the application of heat therapy and/or heat adaptation in this context would be worthwhile.

## Abbreviations

AD	Alzheimer’s disease
ABC	ATP-binding cassette
ADP	Adenosine diphosphate
Akt	Protein kinase B (PKB)
Aβ	β-Amyloid
BBB	Blood brain barrier
COX IV	Cytochrome c oxidase
CNS	Central nervous system
eHSP	Extracellular heat shock proteins
ER	Endoplasmic Reticulum
GGA	Geranylgeranylacetone
GRP	Glucose-responsive proteins
GSIS	Glucose-stimulated insulin secretion
GSK-3	Glycogen synthase kinase-3
HSC	Heat shock cognate
HSFs	Heat shock factors
HSR	Heat shock response
IAPP	Islet amyloid polypeptide
iHSP	Intracellular heat shock proteins
JNK	c-Jun N-terminal kinase
LRP1	Low-density lipoprotein receptor-related peptide 1
NBD	Nucleotide binding domain
NK	Natural killer
pTau	Hyperphosphorylated Tau
sHSP	Small HSPs
T2D	Type 2 diabetes
TLRs	Toll-like receptors
